# Role of oncogenic long noncoding RNA KCNQ1OT1 in colon cancer

**DOI:** 10.32604/or.2023.029349

**Published:** 2024-02-06

**Authors:** GANG LIU, LEI SHI, BIN WANG, ZEHUI WU, HAIYUAN ZHAO, TIANYU ZHAO, LIANGHUI SHI

**Affiliations:** 1 Department of Gastrointestinal Surgery, First Affiliated Hospital of Wannan Medical College, Wuhu, 241001, China; 2 Chongqing Key Laboratory of Oral Diseases and Biomedical Sciences, Stomatological Hospital of Chongqing Medical University, Chongqing, 401147, China

**Keywords:** lncRNA KCNQ1OT1, Colon cancer, HCT116 cells, Tumorigenesis

## Abstract

The role of lncRNA KCNQ1 opposite strand/antisense transcript 1 (KCNQ1OT1) in colon cancer involves various tumorigenic processes and has been studied widely. However, the mechanism by which it promotes colon cancer remains unclear. Retroviral vector pSEB61 was retrofitted in established HCT116-siKCN and SW480-siKCN cells to silence KCNQ1OT1. Cellular proliferation was measured using CCK8 assay, and flow cytometry (FCM) detected cell cycle changes. RNA sequencing (RNA-Seq) analysis showed differentially expressed genes (DEGs). Gene ontology (GO) and Kyoto Encyclopedia of Genes and Genomes (KEGG) pathway enrichment analyses were carried out to analyze enriched functions and signaling pathways. RT-qPCR, immunofluorescence, and western blotting were carried out to validate downstream gene expressions. The effects of tumorigenesis were evaluated in BALB/c nude mice by tumor xenografts. Our data revealed that the silencing of KCNQ1OT1 in HCT116 and SW480 cells slowed cell growth and decreased the number of cells in the G2/M phase. RNA-Seq analysis showed the data of DEGs enriched in various GO and KEGG pathways such as DNA replication and cell cycle. RT-qPCR, immunofluorescence, and western blotting confirmed downstream CCNE2 and PCNA gene expressions. HCT116-siKCN cells significantly suppressed tumorigenesis in BALB/c nude mice. Our study suggests that lncRNA KCNQ1OT1 may provide a promising therapeutic strategy for colon cancer.

## Introduction

In humans, protein-coding genes account for approximately 2% of RNA transcripts. The remaining transcripts are noncoding RNAs, which can be classified into short noncoding RNAs and long noncoding RNAs (lncRNAs). The latter are longer than 200 nucleotides in length and lack an open reading frame (ORF) [1–3]. Although lncRNAs are less abundant than mRNA, they can regulate the expression of protein-coding and noncoding genes and interact with RNA/DNA-binding proteins, transcription factors, and microRNA (miRNA) [4,5]. Currently, only about 200 lncRNAs out of an estimated 60,000–100,000 have been extensively studied, particularly in relation to cancer development and progression [6,7]. Accumulating evidence suggests that targeting oncogenic lncRNAs could have potential applications for cancer treatment [8–10]. Colon cancer is a leading cause of death in China, and additional research is necessary to reveal the underlying mechanisms of cancer development and progression and identify novel targets for advanced therapies.

lncRNA KCNQ1 opposite strand/antisense transcript 1 (KCNQ1OT1) is located on chromosome 11p15.5. KCNQ1OT1 mediates methotrexate resistance in HT29 and Caco2 colon cancer cell lines and promotes oncogenic properties in SW480 and DLD1 colon cancer cell lines [11,12]. With the development of bioinformatics, RNA sequencing (RNA-Seq) can be used to analyze global transcript data to investigate KCNQ1OT1 mediated oncogenicity in colon cancer cells.

Previous studies investigating the effects of silencing lncRNA KCNQ1OT1 in colon cancer cells relied primarily on transient transfection with small interfering RNAs (siRNAs) using Lipofectamine, which may have variable and transient effects. Retroviral systems provide a stable, credible, and long-term solution to investigate the mechanisms by which KCNQ1OT1 contributes to colon oncogenicity, both *in vitro* and *in vivo*.

In the current study, we utilized retroviral vector pSEB61 to establish a stable HCT116-siKCN cell line to explore the downstream genes of KCNQ1OT1 and their contribution to the colon cancer phenotype. Our data demonstrated that KCNQ1OT1 silencing inhibited the growth of HCT116 and SW480 cells and reduced the proportion of cells in the G2/M phase. To validate the downstream genes, we employed RNA-Seq analysis, RT-qPCR, immunofluorescence, and Western blotting. Our findings indicate that lncRNA KCNQ1OT1 plays a critical role in colon cancer development and targeting it may provide a promising option for clinical therapy.

## Methods

### Cell lines and cell culture

The HCT116 cell line was obtained from Procell (CVCL_B8AV, Wuhan, China) and cultured in complete McCoy’s 5A medium (PM150710B, Procell). Human CRC cell lines HCT8 (CVCL_2478), LOVO (CVCL_0399), SW480 (CVCL_0546) and normal colon epithelial cell lines NCM460 (CVCL_0460) was obtained from The Cell Bank of the Chinese Academy of Sciences (Shanghai, China). The 293 Phoenix Ampho (293PA) (CVCL_4W26) and HEK-293 (CVCL_0045) cells were generously provided by Prof. Tong-Chuan HE (University of Chicago, Chicago, USA) and they were cultured in Dulbecco’s Modified Eagle’s medium-supplemented with 10% fetal bovine serum (FBS; Hyclone, CA, USA), as well as 100 units of penicillin, and 100 mg of streptomycin and maintained in an incubator with 5% CO_2_ at 37°C.

### Retrovirus production and stable cell lines

The lncRNA-KCNQ1OT1 siRNA (siKCNQ1OT1) was cloned and designed to be synthesized into the pSEB61 vector (pSEB61-siKCNQ1OT1) by Decoding Therapeutics Corp (Mt Prospect, USA). The retroviral vector pSEB61-siKCNQ1OT1 was developed into a retrovirus within the 293PA cells. Initially, 293PA cells were seeded using 70%–80% density onto 100-mm dishes. Following this, pSEB61-siKCNQ1OT1 and pCL-Ampho vectors were co-transfected into 293PA cells producing a retrovirus that was collected at 12 h intervals. The HCT116 and SW480 cells were infected with packaged retrovirus pSEB61-siKCNQ1OT1 concentrated through PEG8000 (89510, Sigma) at a density of 50%. Through selection with Blasticidin S (0.5 μg/ml), stable knockdown expression of KCNQ1OT1 or control cell lines, assigned as HCT116-siKCN, HCT116 and SW480-siKCN, SW480, were obtained.

### Total RNA extraction and real-time polymerase chain reaction (qPCR)

Total RNA was extracted from the cells using TRIzol Reagents (Invitrogen, Carlsbad, CA, USA) according to the manufacturer’s instructions. Reverse transcription was then carried out to generate cDNA templates, followed by SYBR Green-based qPCR analysis using the thermo cycler CXF-Connect (Bio-Rad, CA, USA). The following thermocycling conditions were used: The reaction mixture was subjected to initial denaturation at 95°C for 10 min, followed by 40 amplification cycles. Each cycle comprised of three steps: denaturation (94°C for 5 s), annealing (60°C for 30 s) and extension (72°C for 35 s), followed by a final extension at 72°C for 10 min. The primers used in this study are shown in Table 1, with internal control using GAPDH.

**Table 1:** List of primers used for qPCR analysis

Gene symbol	Gene name	Primer (5′-3′)
*SKP2*	S-phase kinase associated protein 2	F: TGCTAAGCAGCTGTTCCAGA
		R: AAGATTCAGCTGGGTGATGG
*CCNE2*	Cyclin E2	F: GGGGATCAGTCCTTGCATTA
		R:CCCAGCTTAAATCAGGCAAA
*PCNA*	Proliferating cell nuclear antigen	F: GGCGTGAACCTCACCAGTAT
		R: TTCTCCTGGTTTGGTGCTTC
*ORC6*	Origin recognition complex subunit 6	F: GCAGTGAACATGGCTTCAAA
		R: AGCAGTGCAGCAGAAGTGAA
*TTK*	TTK protein kinase	F: CAGCAGCAACAGCATCAAAT
		R: TGCTTGAACCTCCACTTCCT
*MCM3*	Minichromosome maintenance complex component 3	F: AGCTTCTGCGGTATGTGCTT
		R: CCTGTTTCCTGGTCTGTGGT
*MCM4*	Minichromosome maintenance complex component 4	F: TTGAAGCCATTGATGTGGAA
		R: GGCACTCATCCCCGTAGTAA
*MCM7*	Minichromosome maintenance complex component 7	F: CGGTGCTGGTAGAAGGAGAG
		R: AAACCCTGTACCACCTGTCG
*GAPDH*	Glyceraldehyde-3-phosphate dehydrogenase	F: CAGCGACACCCACTCCTC
		R: TGAGGTCCACCACCCTGT

### RNA isolation, cDNA library construction, sequencing, and data analysis

Total RNA was extracted from HCT116 and HCT116-SiKCN cells using TRIzol reagent (Invitrogen) according to the manufacturer’s instructions. Purification of mRNA from total RNA was achieved using poly-T oligo-attached magnetic beads after assessing RNA quality using NanoDrop2000 (Thermo Scientific, CA, USA).

The raw sequencing data from this study have been deposited in the Genome Sequence Archive (https://ngdc.cncb.ac.cn/gsa/) in BIG DataCenter (https://ngdc.cncb.ac.cn/), Bejing Institute of Genomics (BIG), Chinese Academy of Sciences, under the accession number: HRA004982.

Gene expression was evaluated based on fragments per kilobase of transcript per million fragments mapped (FPKM) values and DEGs were identified using Cuffdiff. Genes were considered significant if they met the following criteria: |log2(fold change)| ≥ 1 and false discovery rate <0.01. To compare the statistical enrichment of DEGs in Gene Ontology (GO) and Kyoto Encyclopedia of Genes and Genomes (KEGG) pathways, the entire genome background was used as a reference.

### Immunofluorescence and immunohistochemical staining

Immunofluorescence and immunohistochemical staining was performed following the manufacturer’s instructions. Culture cells were fixed using paraformaldehyde, followed by permeabilized with 0.1% Triton X-100, and blocking using 10% Block Aid^TM^ (B10710, Invitrogen). The primary antibodies used in this study were B-cell lymphoma 2 (BCL2) (1:200, ab182858, Abcam, CA, USA), proliferating cell nuclear antigen (PCNA) (1:200, ab92552, Abcam, CA, USA), and cyclin E2 (CCNE2) (1:200, ab40890, Abcam, CA, USA). Subsequent to primary antibody incubation, the cells were washed and incubated with 2 µg/mL Donkey Anti-Rabbit IgG (H+L) Highly Cross-Adsorbed Secondary Antibody, Alexa Fluor 594 (A-21207-1, Thermo Scientific, CA, USA) for immunofluorescence staining or for immunohistochemical staining. Following staining, nuclei were counterstained using diamidino-2-phenylindole (DAPI) and the cells were imaged using a laser scanning confocal microscope. Negative controls were stained without primary antibodies or control IgG.

### Western blotting

Total proteins were extracted from the cells, followed by quantification using the RIPA lysis buffer and a BCA detection kit (Beyotime, China), according to the manufacturer’s instructions. Equal amounts of protein were then separated using 10% SDS-PAGE and transferred onto PVDF membranes (Millipore, Billerica, MA, USA). The membranes were then blocked, followed by incubation with primary antibodies against BCL2 (1:1000, ab182858, Abcam), caspase3 (CASP3) (1:1000, 9662s, Cell Signaling Technology), PCNA (1:1000, ab92552, Abcam), CCNE2 (1:1000, ab40890, Abcam), and β-actin (1:1000, ab8226, Abcam) at 4°C for one night. Peroxidase—conjugated secondary antibodies were then used for incubation at room temperature. Chemiluminescence (Beyotime) was used for the detection of the bands, with protein bands quantified using Image J software (http://www.imagej.nih.gov/ij/v2.4.1).

### Cell proliferation assays

Cell proliferation was assessed using the CCK8 assay, specifically the Cell Counting Kit 8 (CCK8) from KeyGen Biotech, China, following the manufacturer’s instructions. HCT116, SW480, HCT116-siKCN and SW480-siKCN cells were seeded into 96-well plates at a density of 2 × 10^3^ cells/well and co-cultured every 24 hours. At 24, 48, 72, 96 and 120 h, 10 μl of the CCK8 reagent was added to each well and mixed uniformly. The cells were then incubated for another three hours before measuring the optical absorbance at 450 nm. Each group was evaluated in five replicate wells, and the experiment was conducted in triplicate.

### Cell cycle distribution assay

HCT116, SW480, HCT116-siKCN and SW480-siKCN cells were seeded into 6-well plates at a density of 2 × 10^6^ cells/well and wait for the cells to grow to 60%–70%, and then the cells were washed with 0.1 M PBS after digestion with 0.25% Trypsin. Next, 1 × 10^6^ cells were fixed using 75% ethyl alcohol at 4°C overnight. The cells were stained with propidium iodide (C1052, Beyotime) at 37°C for 30 min while in the dark, followed by the analysis of cells using a flow cytometer (FACScan) equipped with CellQuest software (both from BD Biosciences, FranklinLakes, NJ, USA).

### In vivo bioluminescence tumor xenograft

Ethical guidelines were followed during all animal studies, as approved by the Ethics Committee of Wannan Medical College (No. 2021012). Ad-FLuc was provided by Prof. Tong-Chuan HE (University of Chicago). HCT116 and HCT116-siKCN cells were infected with Ad-Fluc to produce HCT116-FLuc and HCT116-siKCN-FLuc, respectively. 4–6-week-old BALB/c nude mice (male/female ratio 1:1) were subcutaneously injected with a total of 1.5 × 10^7^ cells per injection were collected and injected subcutaneously into the right dorsal flank of the mice in 200 mL of PBS (4 mice per group) for the tumorigenesis assay. The animals were intraperitoneally injected with luciferase (ab145164, Abcam) after 2 and 14 days of injection, and bioluminescence imaging was performed using the Berthold LB983 imaging system. INDIGO software (Berthold Technologies) was used to analyze the data from bioluminescence images. The mice were euthanized after 20 days of injection and the tumors were collected and analyzed after making paraffin sections. Immunofluorescence staining using anti-PCNA (1:400, ab92552, Abcam) and anti-CCNE2 (1:400, ab40890, Abcam) was conducted to detect and quantify positive cells in three randomly selected high-power fields of each section.

### Statistical analysis

All data are expressed as the mean ± standard deviation. The statistical analysis was performed using SPSS 22.0 software. The statistical significance of the data was evaluated using Student’s *t*-test or one-way analysis of variance. *p* < 0.05 was considered statistically significant.

## Results

### Establishing the HCT116-siKCN stable cell line and the expression of KCNQ1OT1 in CRC tissues and cell lines

**Figure 1:**
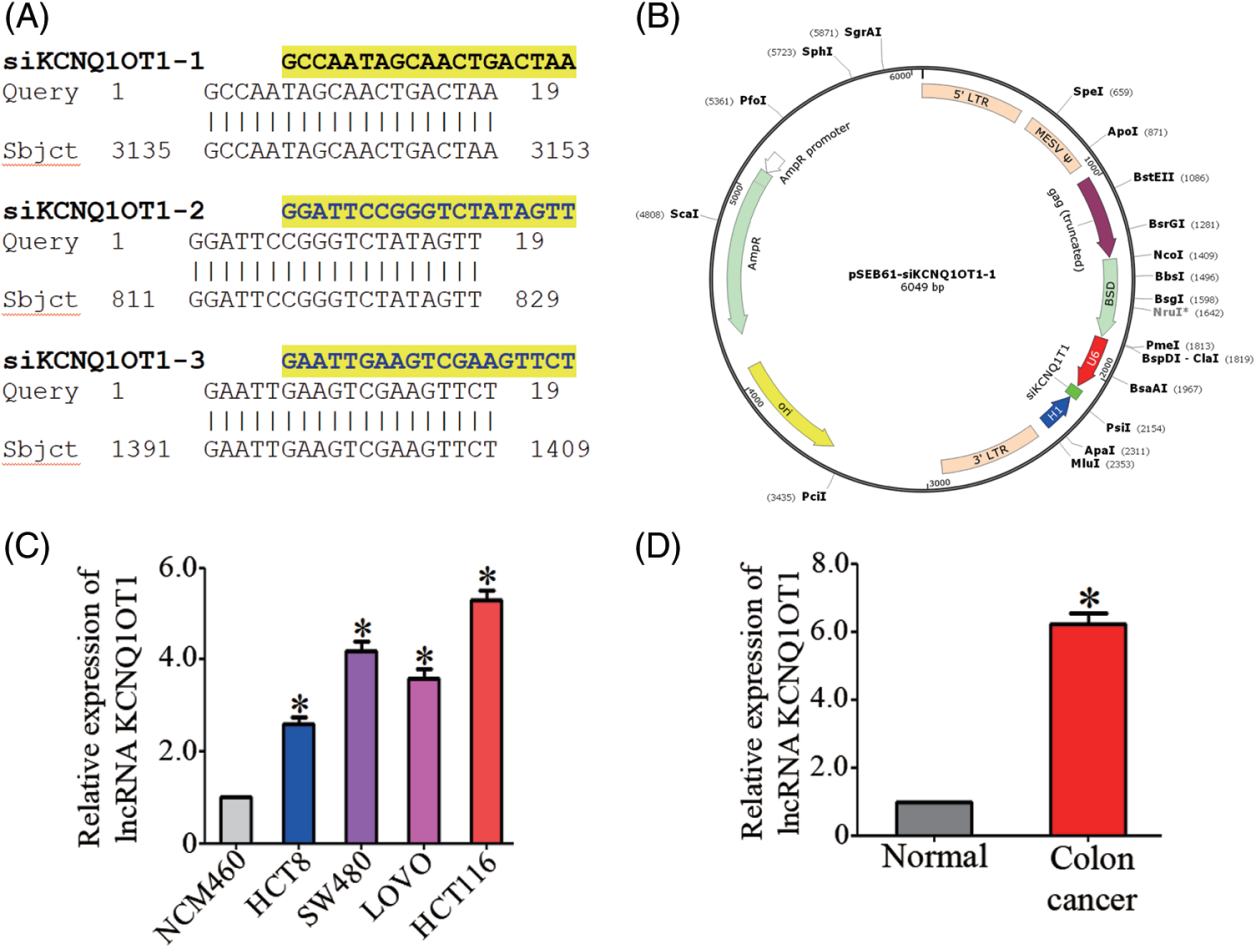
HCT116-siKCN stable cell line with the retroviral vector pSEB61 and the expression of KCNQ1OT1 in CRC tissues and cell lines. (A) Sequence alignment of three lncRNA KCNQ1OT1 siRNAs. (B) Plasmid map showing the introduction of the retroviral vector pSEB61 into the established HCT116-siKCNQ1OT1 cell line. (C) Relative KCNQ1OT1 mRNA expression in normal colon cell (NCM460) and colon cancer cell lines (HCT8, SW480, LOVO and HCT116) measured by real-time PCR. Bar graph data are presented as the mean ± SD; *p < 0.05. (D) Relative KCNQ1OT1 mRNA expression in normal colon cell tissue and colon cancer cell tissue was measured by real-time PCR. Bar graph data are presented as the mean ± SD; *p < 0.05.

The three siRNA sequences are listed in Fig. 1A. Three siRNAs targeting the lncRNA KCNQ1OT1 (NR_002728.3) were constructed using the pSEB61 vector, which contains the U6 and H1 promoters to drive siRNA expression (Fig. 1B). KCNQ1OT1 mRNA levels were assessed in normal colon mucosal epithelial cell line (NCM460) and four CRC cell lines (HCT 8, SW480, LOVO, and HCT116) using qRT-PCR (Fig. 1C).

The mRNA expression of KCNQ1OT1 was significantly higher in CRC tissues compared to adjacent normal tissues (Fig. 1D).

### siRNA-KCNQ1OT1 inhibits cell proliferation and decreases the proportion of cells in the G2/M phase

**Figure 2:**
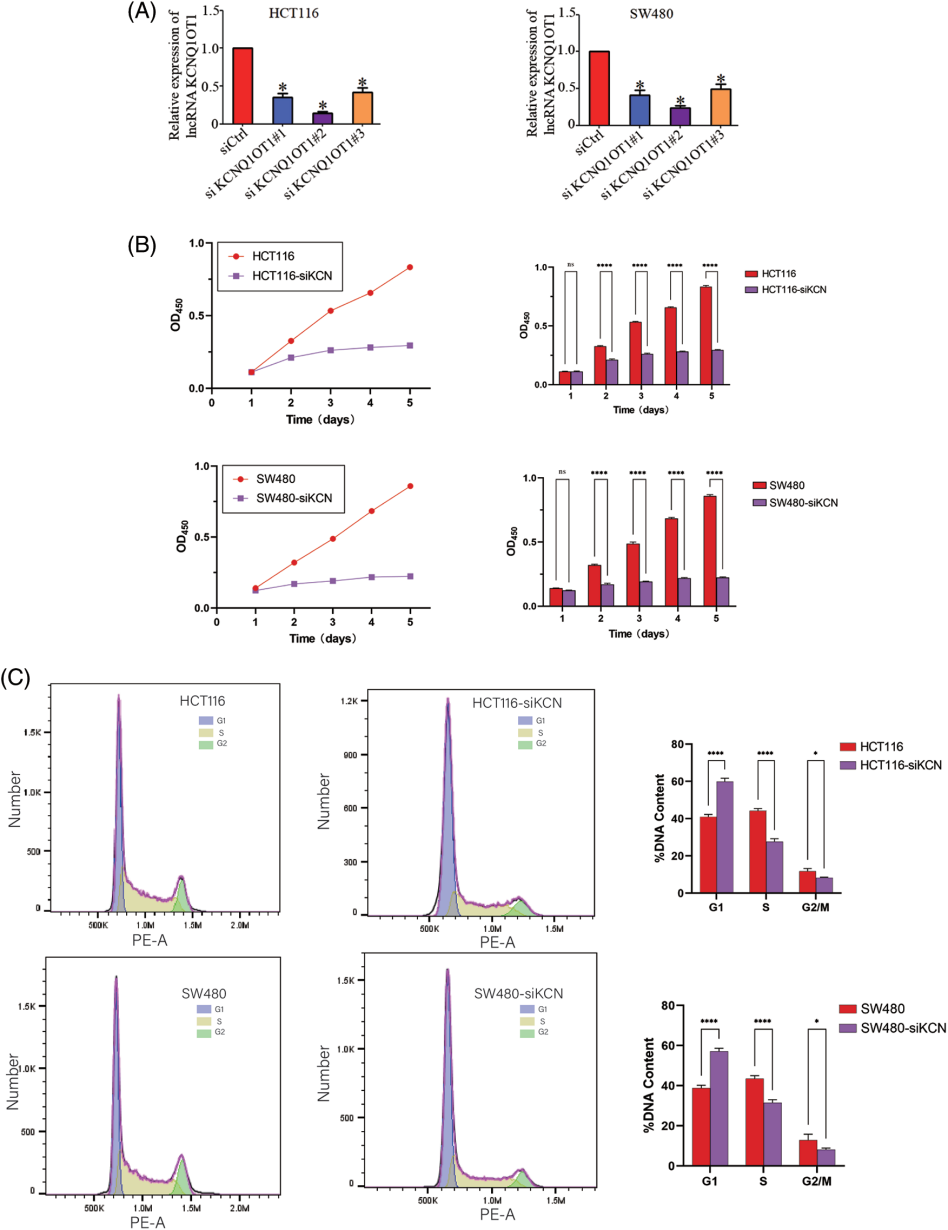
Effects of KCNQ1OT1 silencing on cell proliferation and cell cycle in HCT116 and SW480 cells. (A) Relative KCNQ1OT1 mRNA expression in HCT116 and SW480 cells expressing KCNQ1OT1 siRNA#1, KCNQ1OT1 siRNA#2 and KCNQ1OT1 siRNA#3 analyzed by real-time PCR. Experiments were conducted independently thrice. Bar graph data are presented as the mean ± SD; *p < 0.05. (B) The proliferation capacities were detected by CCK8 assay in CRC cells transfected with the HCT116, HCT116-siKCN, SW480, and SW480-siKCN cells. Experiments were performed independently thrice. Bar graph data are presented as the mean ± SD; *p < 0.05, ****p < 0.0001. Scale bars, 1 cm. (C) Cell cycle progression was assessed using flow cytometry (FCM), the results of which revealed cell cycle arrest at the S/G2/M phase in HCT116, HCT116-siKCN, SW480, and SW480-siKCN cells. Experiments were performed independently thrice; *p < 0.05, ****p < 0.0001.

We validated the transfection efficiency using a PCR experiment (Fig. 2A) and performed CCK8 assay to determine the role of KCNQ1OT1 in HCT116 and SW480 cell proliferation. The CCK-8 cell viability assay showed that knockdown of HCT116 and SW480 cells significantly inhibited the proliferation ability of CRC cells (Fig. 2B). A quantitative assessment of the stained cells indicated that significantly fewer HCT116-siKCN and SW480-siKCN cells appeared at 1, 2, 3, 4 and 5 days than HCT116 and SW480 cells (Fig. 2B). These results showed that the proliferation of HCT116 and SW480 cells were inhibited by siRNA-KCNQ1OT1 transfection. Next, we analyzed the cell cycle using flow cytometry. The proportion of HCT116-siKCN and SW480-siKCN cells in the G2/M phase was significantly decreased (*p* < 0.001) (Fig. 2C). The proportion of cells in the G0/G1 and S phases between HCT116-siKCN and HCT116 as well as SW480-siKCN and SW480 cells did not differ significantly, suggesting that the downregulation of KCNQ1OT1 expression reduced the proportion of cells in the G2/M phase but not in the G0/G1 or S phase.

### Analysis of differentially expressed genes (DEGs) between HCT116 and HCT116-siKCN cells

**Figure 3:**
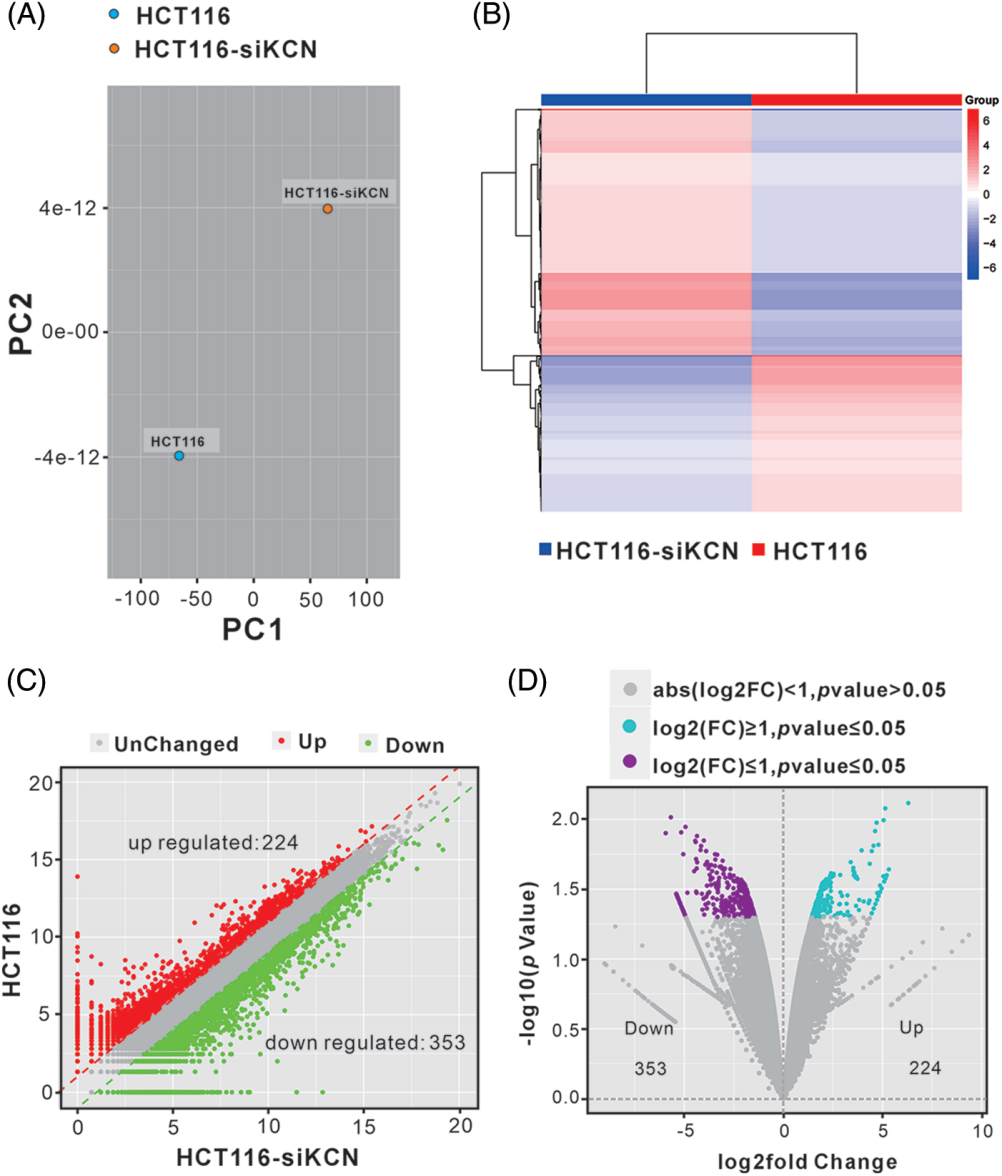
Analysis of differentially expressed genes (DEGs) between HCT116 and HCT116-siKCN cells. (A) The PCA diagram exhibited a similar degree between samples through dimensional reduction. (B) The correlation between HCT116 and HCT116-siKCN cells was determined using a hierarchical cluster heatmap analysis of DEG expression. Red indicates upregulated genes, while blue indicates downregulated genes. (C and D) We found 244 upregulated (red dots) DEGs and 353 downregulated (green dots) DEGs in the two cell lines using scatter (C) and volcano plots (D).

lncRNAs interact with DNA, miRNA, mRNA, and proteins, leading to differential gene expression. To investigate how KCNQ1OT1 affects HCT116 cell proliferation and cycle distribution, RNA-Seq and bioinformatic analyses were conducted. Known genes were calculated using FPKM, with 12906 genes obtained in HCT116 cells and 13032 genes in HCT116-siKCN cells (FPKM ≥ 1) after quality control. However, more than 60% of the genes had an FPKM less than 1. The A PCA diagram was used to determine the degree of similarity between the samples (Fig. 3A) while a heatmap highlighted mRNA expression differences between the two cell lines (Fig. 3B). Using fold-change filtering (|log2(fold change)| > 1), and Student’s *t*-test (*p* < 0.05), we identified 577 DEGs. The significant DEGs between HCT116 and HCT116-siKCN cells were visualized using a scatter plot (Fig. 3C), and Volcano plot (Fig. 3D) with 353 DEGs downregulated and 224 DEGs upregulated in HCT116 cells compared to HCT116-siKCN cells.

**Figure 4:**
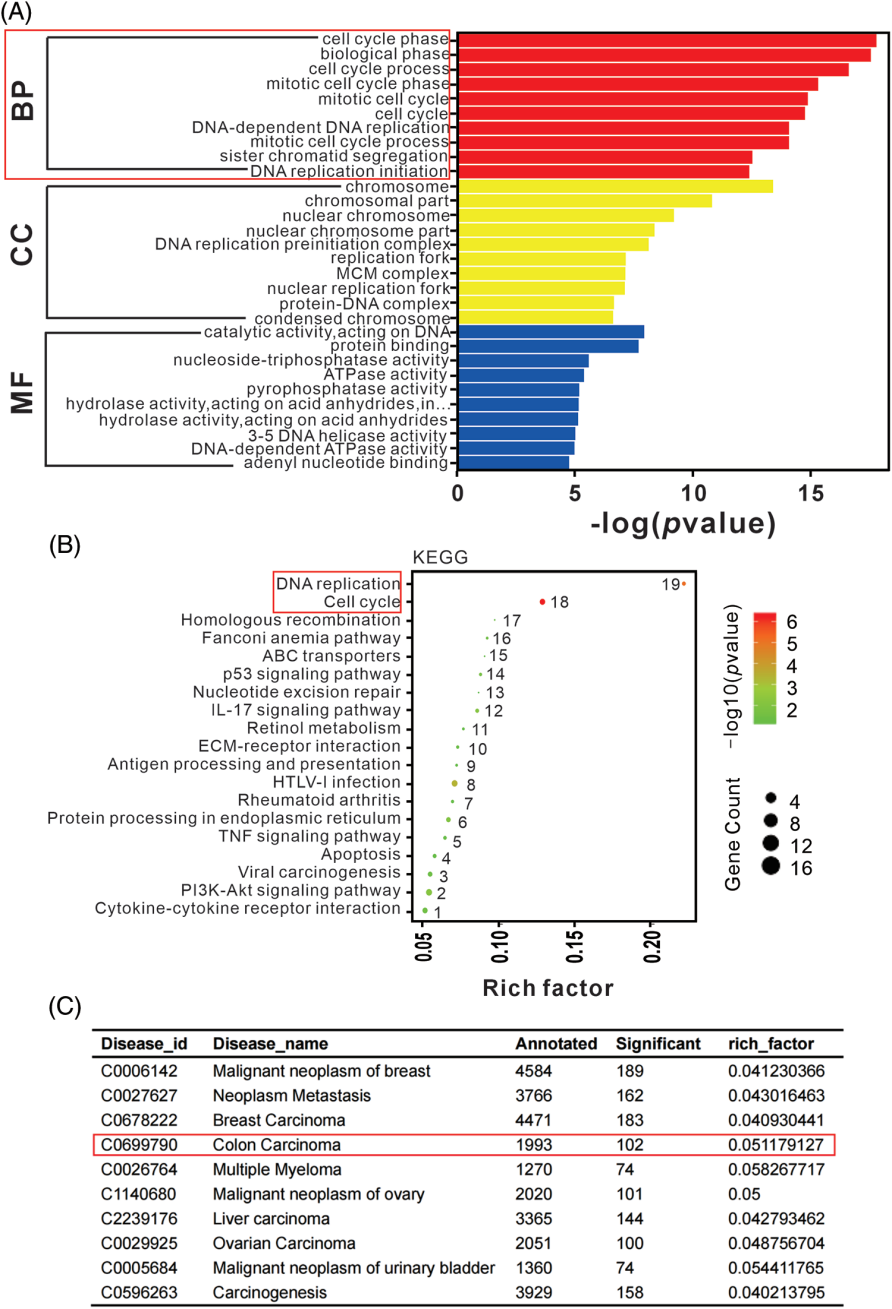
Gene ontology (GO), Kyoto Encyclopedia of Genes and Genomes (KEGG), and disease enrichment analysis results of differentially expressed genes (DEGs) using RNA sequencing. (A) GO term enrichment analysis of DEGs. The bar plot indicates enrichment scores of the top ten genes significantly enriched in biological processes, cellular components, and molecular functions. (B) Top 19 signaling pathways identified in KEGG enrichment pathway analysis of DEGs. (C) Top ten disease enrichment pathways of lncRNA-KCNQ1OT1. In total, 102 DEGs were identified in colon carcinoma.

Functional analysis of DEGs was performed to evaluate the observed differences. The enrichment of DEGs in different gene ontology (GO) functions were analyzed, with the top 20 GO terms suggest significant enrichment in various functions related to DNA replication, chromosome segregation, and cell cycle (Fig. 4A). KEGG pathway analysis revealed that significant DEGs were mainly associated with functions such as cell cycle, DNA replication, and various signaling pathways (Fig. 4B). Disease enrichment analysis showed that lncRNA-KCNQ1OT1 was associated with several cancer treatments (Fig. 4C). Specifically, 102 DEGs were identified in colon carcinoma.

### Validation of downstream targets of DNA replication and cell cycle pathways

**Figure 5:**
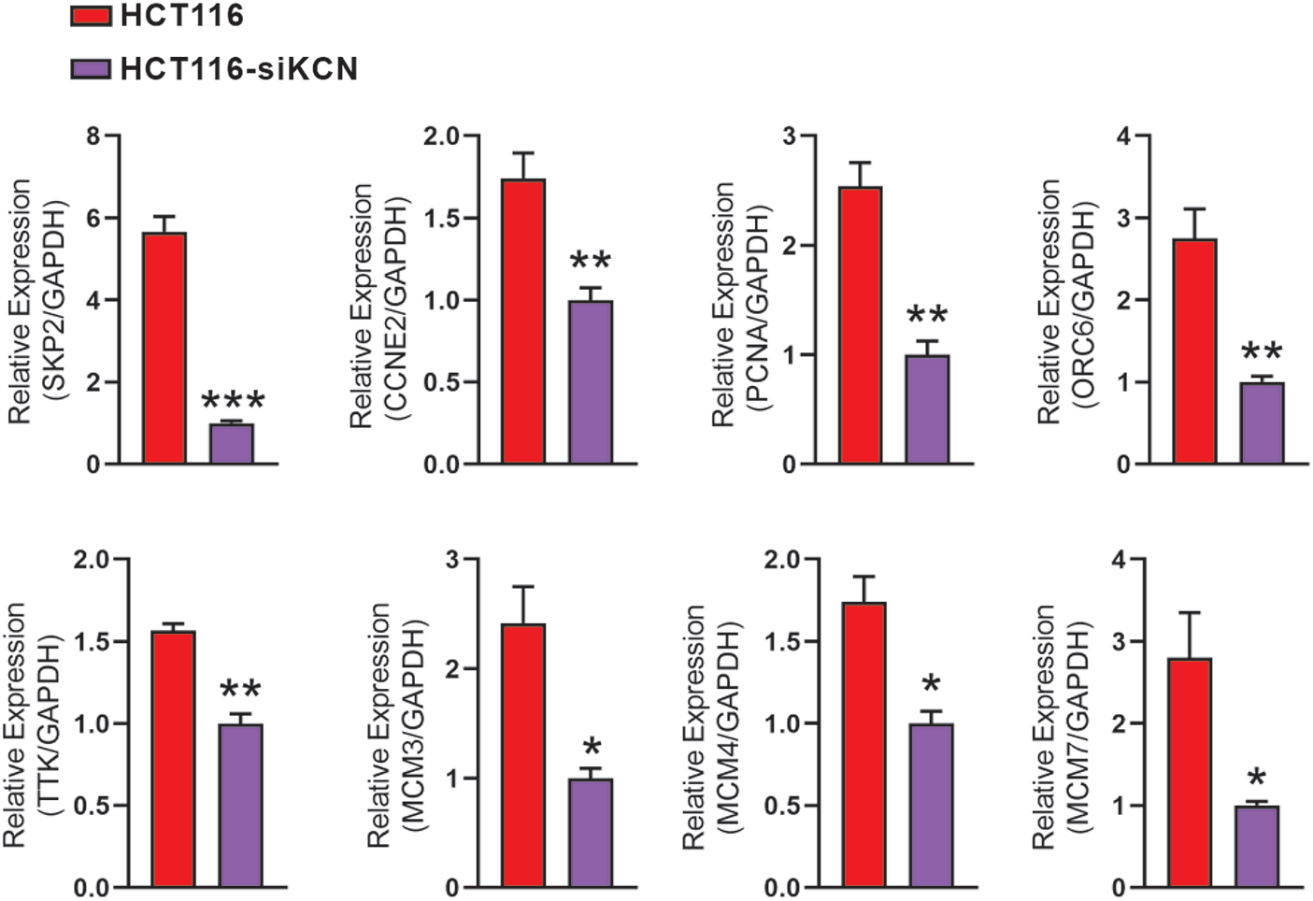
Validation of downstream genes involved in DNA replication and cell cycle pathways using RT-qPCR. RT-qPCR performed using primers for SKP2, CCNE2, PCNA, ORC6, TTK, MCM3, MCM4, MCM7, and GAPDH. Expression of PCNA, SKP2, ORC6, TTK, MCM3, MCM4, MCM7, and CCNE2 decreased in HCT116-siKCN cells. Each assay was performed in triplicate and compared with the corresponding HCT116 cell control group, *p < 0.05, **p < 0.01, and ***p < 0.001.

For RT-qPCR-based validation, we identified 19 DEGs enriched in DNA replication and cell cycle pathways as shown in Fig. 5. Subsequent quantitative analysis confirmed a decrease in the expression of PCNA, SKP2, ORC6, TTK, MCM3, MCM4, MCM7, and CCNE2 in HCT116-siKCN cells.

**Figure 6:**
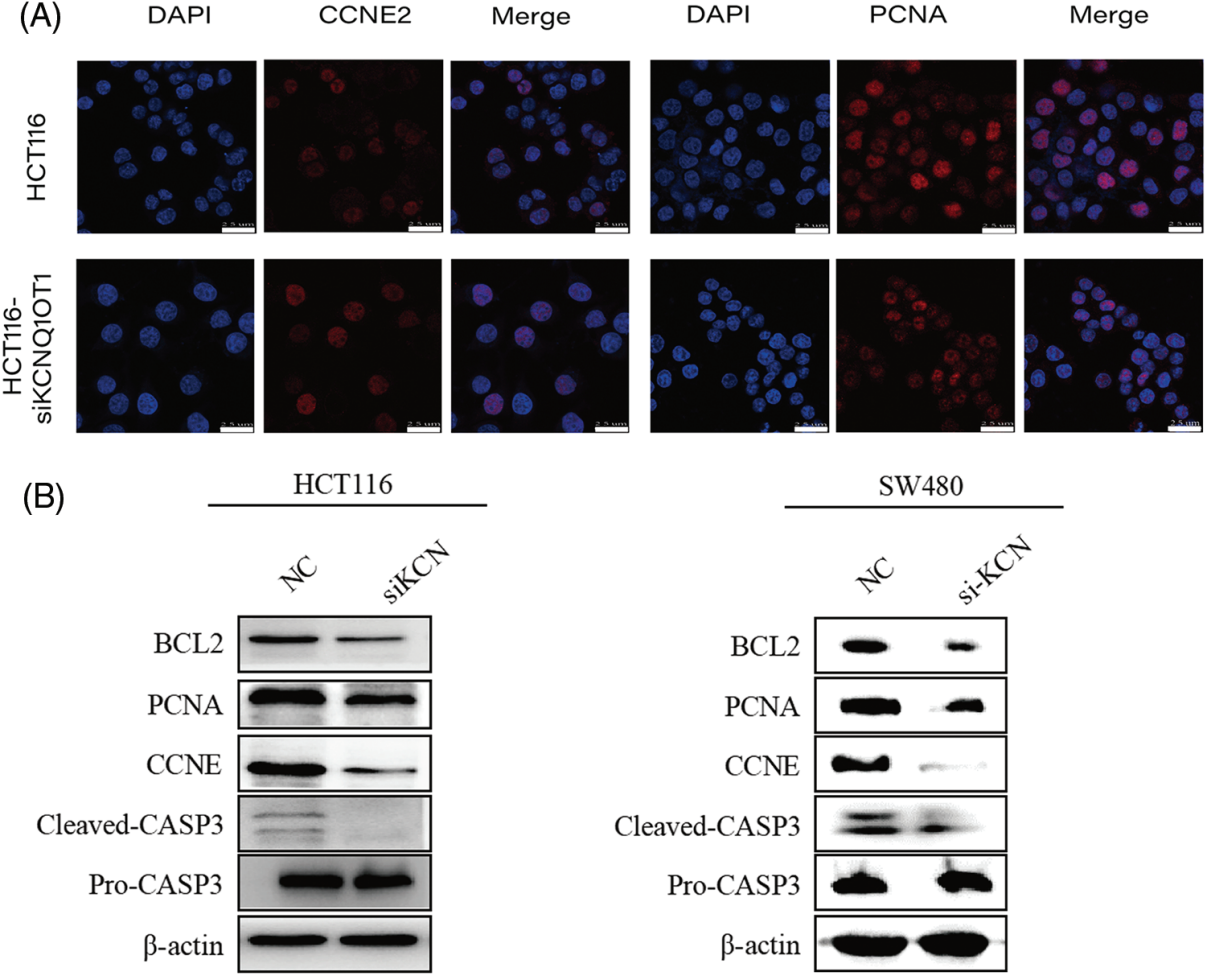
Validation of downstream targets involved in DNA replication and cell cycle pathways using immunofluorescent staining and western blotting. (A) Immunofluorescent staining (red) results indicated that CCNE2 and PCNA exhibited strongly positive expression in the nucleus. Nuclei were stained blue using DAPI. Bar = 25 µm. (B) Western blotting results showing CCNE2, PCNA, BCL-2, and CASP3 expression in two groups in HCT116, HCT116-siKCN, SW480, and SW480-siKCN cells. β-Actin was used to confirm equal loading.

Of these, CCNE2 and PCNA have been identified as key targets of KCNQ1OT1 regulators for promoting cell proliferation in breast cancer and glioma. To further examine protein localization in HCT116 and HCT116-siKCN cells immunofluorescence staining was performed. The results showed high expression of PCNA and CCNE2 in the nucleus as illustrated in Fig. 6A. However, positive cell counts did not differ significantly between the two cell lines. Western blotting was performed to assess protein expression which revealed a decrease in the expression of CCNE2 and PCNA, as shown in Fig. 6B.

Previous studies have reported reduced expression of the anti-apoptotic protein, BCL2, and elevated expression of the apoptotic effector CASP3 in SW480 and LS1034 cells. Our Western blotting results demonstrated reduced BCL2 and cleaved CASP3 expression, with no significant difference was observed in pro-CASP3 expression in HCT116-siKCN and SW480-siKCN cells, as illustrated in Fig. 6B.

### siRNA-KCNQ1OT1 ablates cellular vitality and tumor xenograft in vivo

**Figure 7:**
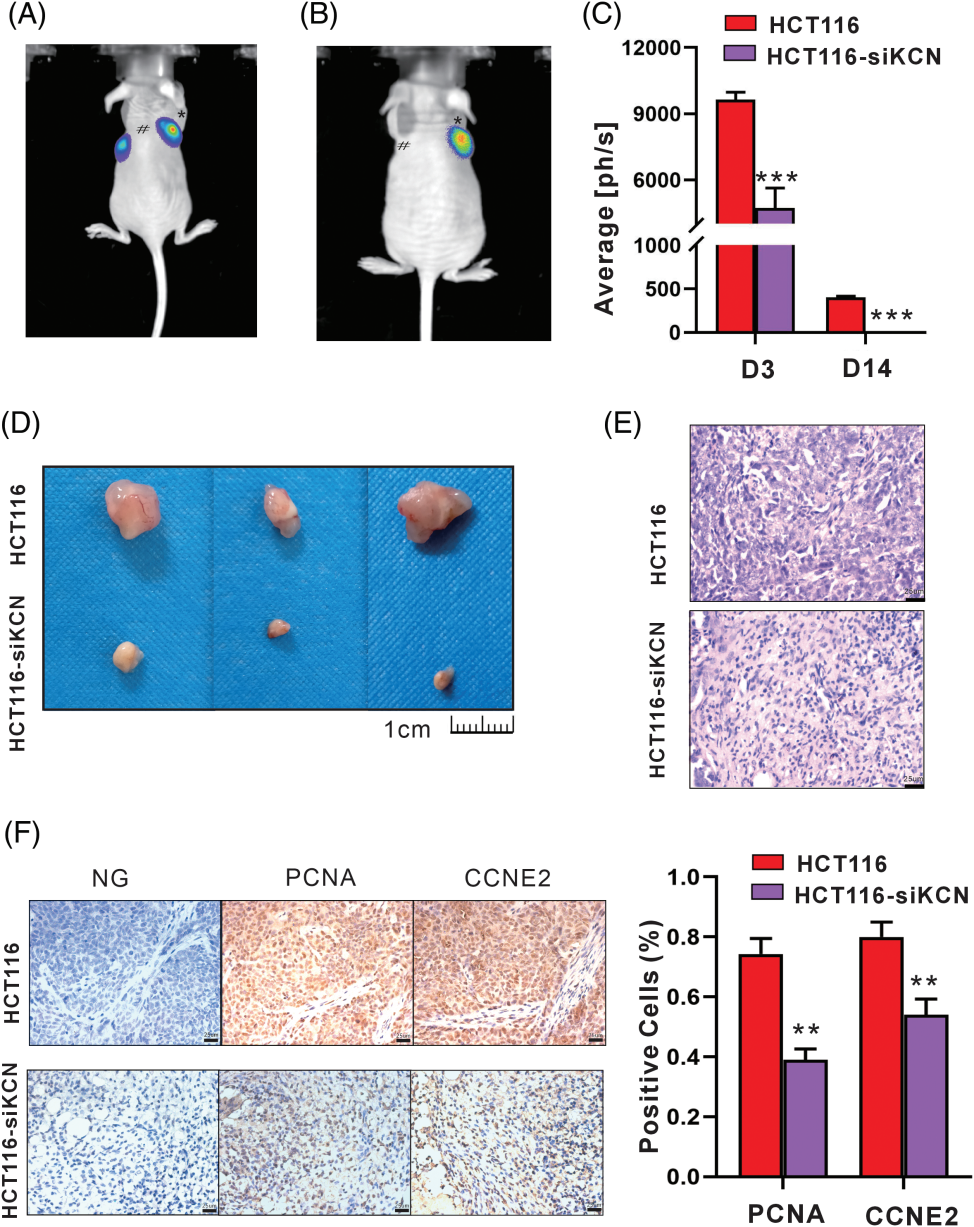
siRNA-KCNQ1OT1 ablates cell vitality and tumor xenograft *in vivo*. Tumor growth bioluminescence imaging was performed during the peak time point after the intraperitoneal injection of 140 mg/kg d-fluorescein sodium salt on days 3 (A) and 14 (B) of cell inoculation. The imaging exposure time was 60 seconds. # Represents HCT116 cells, while * represents HCT116-siKCN cells. (C) Images of subcutaneous tumors of HCT116 and HCT116-siKCN group mice on day 20 after injection. (D) Images of the corresponding xenografts in the HCT116 and HCT116-siKCN groups (*t*-test, ***p < 0.001). (E) Hematoxylin and eosin staining of sections from tumor xenograft. Bar = 25 µm. (F) CCNE2 and PCNA staining and quantification of positive cells. Bar = 25 µm (*t*-test, **p < 0.01).

Finally, we established a xenograft model using HCT116-FLuc and HCT116-siKCN-FLuc cells to demonstrate the role of KCNQ1OT1 in cellular vitality and tumorigenesis *in vivo*. Bioluminescence images showed readily detectable in both groups on day 2 after injection, while signals disappeared in HCT116-siKCN-FLuc-injected mice by day 14. Compared to HCT116-FLuc-injected mice, the bioluminescence signals in HCT116-siKCN-FLuc-injected mice decreased significantly by day 14, as shown in Figs. 7A–7C. After 20 days injection, the mice were sacrificed, and the tumors were collected for further measurement.

Tumor xenograft volume was significantly reduced in the HCT116-siKCN group by day 14 and almost disappeared at day 30 after injection as shown in Figs. 7D and 7E. Finally, immunohistochemical staining of sections from the tumor xenografts revealed a decrease in the proportion of both PCNA- and CCNE2-positive cells were decreased in the HCT116-siKCN group, as shown in Fig. 7F.

## Discussion

In this study, we successfully established HCT116-siKCN and SW480-siKCN cells with downregulated expression of the lncRNA KCNQ1OT1, as compared to the HCT116 and SW480 cells. Prior evidence suggests that the downregulation of KCNQ1OT1 expression in HCT116 cells may suppress cell proliferation, decrease the proportion of cells in the G2/M phase *in vitro*, as well as significantly reduce tumor size and cellular vitality in ectopic xenografts in nude mice. To further explore the downstream genes, we analyzed and validated DEGs by summarizing the global transcript data of the two cell lines. Our results suggest that silencing KCNQ1OT1 in HCT116 and SW480 cells could suppress tumor progression.

Colon cancer is among the top five most commonly diagnosed digestive cancers in China [13]. Noncoding RNAs(lncRNAs), which are over 200 nucleotides in length, and can function as tumor suppressors or oncogenic regulators indifferent cancers, including colon cancer [8,14–16]. The abnormal expression of lncRNAs presents them as promising diagnostic or prognostic biomarkers and therapeutic targets for colon cancer. Approximately 200 differentially expressed lncRNAs have been identified in colon cancer patients involved in patient outcomes and drug resistance, have been obtained according to The Cancer Genome Atlas [13,17]. However, the role of KCNQ1OT1 in colon cancer remains incomplete [18,19]. It has been found that high levels of lncRNA KCNQ1OT1 are associated with poor prognosis in CRC patients and are critical for CD8+ T cell function in CRC. These findings suggest that lncRNA KCNQ1OT1 is an important immunotherapeutic target that may enhance CD8+ T cell response in CRC [20]. Thus, this study aimed to explore the molecular mechanisms through which KCNQ1OT1 is involved in colon cancer tumorigenesis by performing a global bioinformatics analysis of downstream genes.

Previous studies have indicated that lncRNA KCNQ1OT1 participates in regulating cell proliferation and cell cycle, which contributes to the cancer phenotype [21–24]. In this study, we used CCK8 essay and found that silencing KCNQ1OT1 in HCT116 and SW480 cells inhibited their growth. We analyzed cell cycle distribution by flow cytometry and found that the proportion of HCT116-siKCN and SW480-siKCN cells in the G2/M phase decreased significantly. Thus, we confirmed KCNQ1OT1’s role in influencing colon cancer.

lncRNAs, which lack protein-coding capacity, regulate mRNA processing both inside and outside the nucleus [25,26]. By serving as a competing endogenous RNA (ceRNA), they can regulate miRNA expression and target downstream molecules [13]. Therefore, we evaluated the molecular mechanisms through which KCNQ1OT1 affects the viability of HCT116 cells using RNA-Seq. To overcome the effects of a serum-based culture medium on cell proliferation, FBS was introduced into the medium at a low concentration of 2 h before RNA isolation. We identified 577 DEGs, and GO terms analysis indicated that DNA replication and mitotic cell cycle phase were enriched. Additionally, KEGG pathway analysis revealed that downregulated DNA replication and cell cycle-associated DEGs were enriched in HCT116-siKCN cells. Furthermore, disease enrichment demonstrated that 102 DEGs were associated with colon carcinoma.

After screening for downregulated DNA replication- and cell cycle-associated DEGs, we validated 19 genes using qPCR assay. The results of this assay revealed decreased expression of PCNA, SKP2, ORC6, TTK, MCM3, MCM4, MCM7, and CCNE2 in HCT116-siKCN cells. Additionally, western blotting results confirmed the significant downregulation of CCNE2 and PCNA expression in HCT116-siKCN cells. The roles of BCL2 family members in B-cell lymphoma-associated apoptosis were discovered in the 1980s [27], with BCL2 exerts anti-apoptotic effects that promote cancer development and progression. The BCL2 inhibitor, Venetoclax, induces apoptosis and could potentially be used to treat various cancers, such as chronic lymphocytic leukemia [28]. Targeting BCL2 directly is a valid chemotherapeutic strategy; however, understanding its molecular pathway of action would enable us to modify, manipulate, or mimic clinical treatments [29]. Reduced BCL2 expression was observed in HCT116-siKCN and SW480-siKCN cells in the present study. CASP3 is activated, while BCL2 expression is attenuated in tumor cell death [30]. Caspase-dependent intrinsic apoptosis inhibits BCL2 expression, and the reactivation of pro-apoptotic Bax and Bak proteins irreversibly trigger apoptosis [31,32]. Therefore, the expression of CASP3 was detected using western blotting to further explore whether caspase-dependent intrinsic apoptosis occurs in HCT116-siKCN and SW480-siKCN cells, and the expression of cleaved CASP3 was also found to be reduced in these cells. Interestingly, Although we reviewed the RNA-Seq data, we failed to find apoptosis-related DEGs. Thus, the role of CASP3 in KCNQ1OT1 regulation in HCT116 and SW480 cells requires further determination.

Li et al. reported that lncRNA KCNQ1OT1 targeted miR-34/Atg4B to induce KCNQ1OT1-mediated protective autophagy and chemoresistance in osteosarcoma [11]. BCL2 also plays an anti-autophagy role in cancer cells. Further research is necessary to explore the effects of autophagy on HCT116-siKCN cells. High KCNQ1OT1 expression is associated with poor overall survival in colon cancer patients [33]. Wang et al. found that X-box binding protein 1, an important transcription factor that accelerates tumor growth, may downregulate lncRNA KCNQ1OT1 expression in HCT116 cells [34]. Therefore, investigating the adjacent genes regulated by lncRNA KCNQ1OT1 is necessary to determine targeted treatments for colon cancer.

The retroviral vector, pSEB61, was constructed by Prof. Tong-Chuan HE (University of Chicago), and its effectiveness has been previously verified [35]. In this study, we designed and packaged three retroviral vectors for pSEB61-siKCNQ1OT1 into 293PA cells, aiming to silence lncRNA KCNQ1OT1 in HCT116 and SW480 cells. HCT116-FLuc and HCT116-siKCN-FLuc cells were obtained after Ad-Fluc transfection *in vivo* bioluminescence images showed similar vitality of cells implanted in BALB/c nude mice. We observed a significant decrease in the size of tumor xenografts injecting HCT116-siKCN cells. Interestingly, tumor xenografts were almost non-existent at day 30 after implantation suggesting that silencing KCNQ1OT1 using pSEB61 retroviral vector may be a promising option for colon cancer therapy. Our study comprehensively analyzed the DEGs through bioinformatics and found scarce oncogenicity through tumor xenograft silencing KCNQ1OT1 in HCT116 cells. However, further studies are required to investigate the therapeutic effects of using vectors with silencing KCNQ1OT1 in the colon cancer model.

In conclusion, our study indicates that silencing KCNQ1OT1 in HCT116 and SW480 cells may suppress colon cancer progression by negatively regulating DNA replication and the mitotic cell cycle phase-related pathways, targeting the downstream PCNA and CCNE2. KCNQ1OT1 could be a promising target for the treatment of colon cancer.

## Data Availability

All data generated or analyzed during this study are included in this article.
